# Estimation of the Driving Style Based on the Users’ Activity and Environment Influence

**DOI:** 10.3390/s17102404

**Published:** 2017-10-21

**Authors:** Mikhail Sysoev, Andrej Kos, Jože Guna, Matevž Pogačnik

**Affiliations:** Laboratory for Telecommunications, Faculty of Electrical Engineering, University of Ljubljana, Tržaška cesta 25, Ljubljana 1000, Slovenia; mikhail.sysoev@ltfe.org (M.S.); andrej.kos@fe.uni-lj.si (A.K.); matevz.pogacnik@fe.uni-lj.si (M.P.)

**Keywords:** aggressive driving, user environment data, activity data, driving style prediction

## Abstract

New models and methods have been designed to predict the influence of the user’s environment and activity information to the driving style in standard automotive environments. For these purposes, an experiment was conducted providing two types of analysis: (i) the evaluation of a self-assessment of the driving style; (ii) the prediction of aggressive driving style based on drivers’ activity and environment parameters. Sixty seven h of driving data from 10 drivers were collected for analysis in this study. The new parameters used in the experiment are the car door opening and closing manner, which were applied to improve the prediction accuracy. An Android application called *Sensoric* was developed to collect low-level smartphone data about the users’ activity. The driving style was predicted from the user’s environment and activity data collected before driving. The prediction was tested against the actual driving style, calculated from objective driving data. The prediction has shown encouraging results, with precision values ranging from 0.727 up to 0.909 for aggressive driving recognition rate. The obtained results lend support to the hypothesis that user’s environment and activity data could be used for the prediction of the aggressive driving style in advance, before the driving starts.

## 1. Introduction

There is a significant trend in the Internet of Things (IoT) field [1] and computer science towards the development of systems that learn what their users do and then try to model and predict a user’s behavior. The goal of such systems is to acquire the ability to respond in a more adaptive way or provide personalized feedback and actions to their users. These smart services are becoming even more popular and present due to the ubiquity of computers and smartphones, as a lot of such services were and continue to be provided as smartphone applications.

The research in this article demonstrates new approaches to solving the problem of the prediction of the driving style, showing promising results. A new prototype system for collecting users’ environment influence and activity data, heart rate activity and car door opening/closing data was developed, and the results show success in the prediction of the driving style. It should be noted that the term “users’ environment influence and activity data” in this article describe users’ activities such as phone calls made, SMS’ sent and environmental parameters such as ambient noise or light for example and does not assume any formal modeling of user’s context using ontologies or similar.

The initial assumption was that data, collected via smartphone sensors, could be used as a marker for the estimation of the driving style and checked this hypothesis during the research. It should be noted, that the current status of the world road safety remains an ongoing concern. The Global Status Report on Road Safety 2015, reflecting information from 180 countries [2], the UN World Health Organization (WHO) declares that:

- The total number of road traffic deaths remains unacceptably high at 1.25 million per year;
- More than half of road traffic deaths in Europe are car occupants;
- In addition to deaths on the roads, up to 50 million people incur nonfatal injuries each year as a result of road traffic accidents;
- Globally an estimated 3% of gross domestic product (GDP) is lost due to road traffic deaths and injuries.

Road traffic injures are the leading cause of death among young people aged between 15 and 29 years [2]. During driving, when drivers are experiencing a high level of stress situations leading into possibly aggressive driving, the automatic management of different applications may be desired (e.g., calls, SMS, messengers, navigation aids). For this task, the day to day driving provides a good platform for long term measurements of driver’s activities, using data collected before and during driving.

Therefore, the focus of the article is the study of the hypothesis that it is possible to predict the driving style based on the driver’s activities and environment data (which can include physiological parameters of the driver) before and at the very beginning of driving. Additionally, an innovative approach, based on the car door opening and closing (acceleration data) was used in the estimation of the driving style estimation or in the drivers’ stress level recognition. To the best of our knowledge, no such research has been conducted, using this kind of approach.

The remainder of the paper is organized as follows: in Section 2 related work is presented. Section 3 describes the proposed approach for driving style estimation and the experiment procedure. The results are presented in Section 4. Finally, key conclusions are given in Section 5.

## 2. Related Work

A number of approaches based on different methods regarding the evaluation and prediction of the driving style exist. The most common methods for the estimation of the driving style are described below.

### 2.1. Drivers’ Self-Reports and Questionnaires

Several types of questionnaires have been used in this domain: (i) in 1990, the Driving Behaviour Questionnaire (DBQ) was developed to estimate abnormal driving behaviour, [3]; (ii) DBQ was applied for the driver’s anger assessment, impulsiveness and aggressiveness [4]; (iii) a self-report for measuring of unsafe driving practices [5]; (iv) self-reports to identify drivers who potentially act dangerously on the road [6]; and others [7,8,9,10]. The self-reporting approaches suffer from some disadvantages, which makes it difficult to obtain reliable information of the driving style. Namely, the time required to fill the questionnaire is approximately 30 min (e.g., in [4]), which can be inconvenient for the users participating in the survey. Filling the questionnaire after driving can also cause non-objectivity because the results depend on the memory, attention and mood of the driver. Furthermore, the drivers may experience cognitive distortions or succumb to the desire to embellish their true driving style.

The ability to reduce or even avoid the time gap between the occurrence of the event and its assessment can be provided through voice assessments of the situations [11,12]. This approach allows for describing/assessing a specific situation on the road during driving, but it cannot completely replace the questionnaires. It is considered unsafe to ask the driver to fill out the questionnaire or provide voice assessment of the situation during driving as it could lead to the shift of the driver’s attention from the road conditions as a result of increased cognitive load.

### 2.2. Simulation Scenarios

The driving style evaluation in the simulation scenarios in the controlled environment [13,14,15,16,17] is one of the most common methods for studying the driver’s behaviour. Driving simulators allow for the design of various driving situations, including the dangerous ones: human-human dialogues [18], talking by phone [19], driver’s fatigue and highway driving [20], improving safety at railway level crossings [21], etc. Driving simulation approach is well suited for safe testing of various hypotheses and assumptions, testing of which in the real-life driving situations may be unsafe. It is important to note, that the driving style and manoeuvres in the simulation scenarios in controlled environment may be different from the ones in the real-world driving environment [22].

### 2.3. Experts’ Assessment of the Driver’s Style

Driving instructors, sitting in the car during tests or watching videos taken by the front car camera, can evaluate the driving style and driver’s emotional state [23] and then provide a feedback. The disadvantages of this method are relatively high expenses related to the experts’ involvement as well as their subjective evaluations, which may vary from one expert to another. This category also includes more unusual methods for the driver’s style assessment, such as the analysis of driver’s skills based on the evaluation of the passenger’s comfort [24].

### 2.4. Driving Data Analysis

Due to the progress in the field of mobile sensing technologies and growing technical capabilities of these devices, it is possible to collect a variety of objective data about the car and the driving style (e.g., speed, acceleration, braking, shift gear state, engine RPM, fuel consumption, manoeuvres, location, etc.). Such examples of sensing devices, which can be used for drivers’ style recognition, are: On-Board Diagnostic (OBD) [25], inertial sensors on head-mounted devices (HMDs) such as the Google Glass, and specialized mobile devices to identify driving activities and unsafe driving [20], accelerometers (ACC) [26], GPS receivers [27], magnetometers [27], gyroscopes [28], smartphones [29,30], cameras [31], etc.

According to one study [32], it is possible to identify the driver through the analysis of parameters related to their driving such as acceleration and car pedal pressures. Such information can be received not only from car data related to the pedal pressures but also from the analysis of the GPS and the accelerometer data. 

### 2.5. Methods Using the Drivers’ Physiological and Environment Parameters

Some of the approaches are using the drivers’ environment and behavioral data and have been successfully applied in the stress recognition research [33,34,35,36], which is also related to changes in the driving style. A lot of research has also been done in the field of the estimation of different drivers’ conditions such as vigilance, stress, drunkenness, fatigue, driver distraction, by using different wearable sensors. Typical data measurements include the heart rate variability (HRV), electroencephalogram (EEG), electrodermal activity (EDA), electromyography (EMG), photoplethysmogram (PPG), respiration rate and other physiological parameters [23,37,38,39]. It should be noted that wearing sensors for collecting physiological data on the body can provide inconvenience for the drivers. In order to make the measurements more non-invasive, additional methods for determining physiological parameters of drivers were applied, including analysis of video captured from the cameras installed inside and outside the vehicle [40,41,42]. Fatigue and vigilance of drivers were also estimated based on the monitoring of the glance direction, eye closure duration, blink frequency, face position, head rotation and movement of hands [43,44].

## 3. Experiment Setup and Procedure

One of the goals of this study was the prediction of the driving style based on the analysis of the drivers’ activity and environmental data. The initial set of parameters was selected based on related research, described in Section 2 [33,34,35,37]. New parameters for measuring changes in the drivers’ behaviour such as the deviation in the manner of opening and closing of the car door (different speed/intensity of opening and closing the car door, time between opening the car door and driving start) and collecting the data before and during the driving were applied, based on the assumption that they might be relevant for the study. Next, we describe methods for collecting different data types, which were used for the prediction of the driving style.

### 3.1. User Environment Data Collection Using the Smartphone

**Figure 1:**
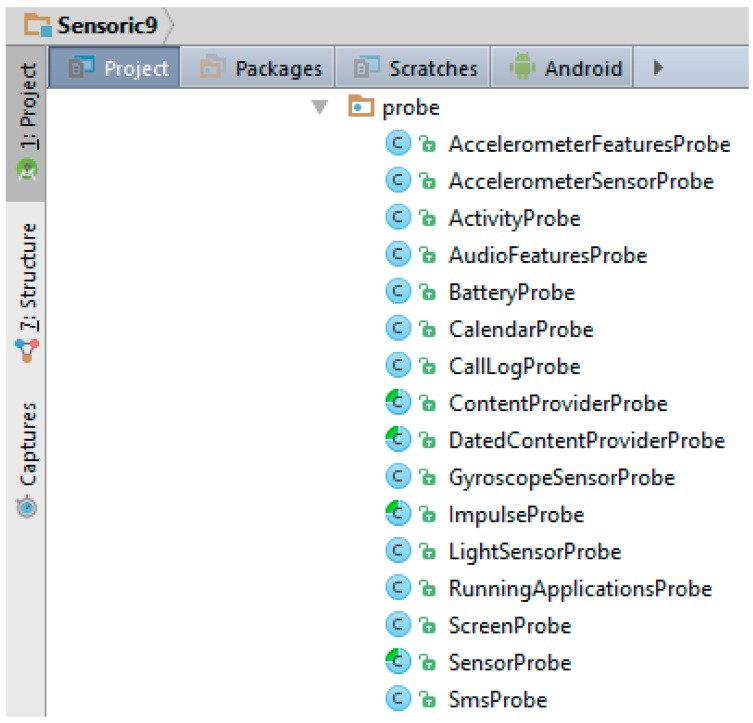
Smartphone sensor data, which were collected via Android application.

Using a smartphone, the following environmental features were collected for further analysis: screen on/off state, audio features (environment noise), light environment features, different gyroscope and accelerometer data, call and SMS log, user’s activity collected through Google activity recognition (GAR) and calendar entries. For that purpose an Android application named *Sensoric* was developed to collect the low-level smartphone data about the current type of activity, as shown in Figure 1.

The presented data features were selected for the driving style analysis based on previous research and under the condition that the smartphone could run without charging for at least one full day. Once all data was collected, it was imported to the MySQL database (DB). Each data type was put in a separate table with the key field “Time” (time in Unix format), which corresponds to the time of the event. Then, after pre-processing, the data was prepared for the analysis and feature selection was made for machine learning tasks.

### 3.2. Heart Rate Data

**Figure 2:**
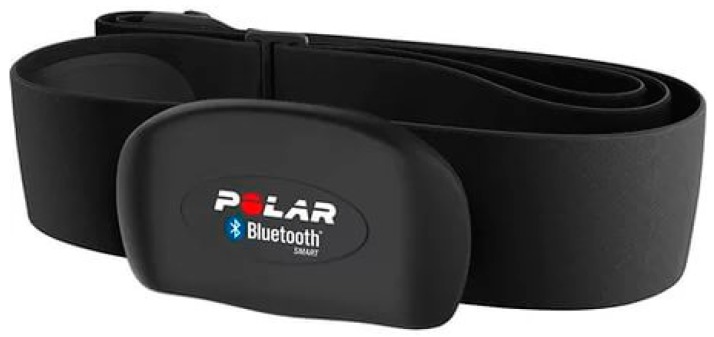
Polar H7 chest belt to collect heart rate data.

To collect inter-beat intervals (RR-intervals) and beats per minute (bpm), the Polar H7 chest belt was used (Figure 2). This heart rate sensor needs Bluetooth 4.0 to connect to the smartphone. To receive and store the HR data, the SelfLoops application [45] was used.

In this research, we used the derivatives from the HRV (heart rate variability) data—SDNN (time domain methods): the standard deviation of NN intervals or “RR variability”. The SDNN approach was chosen because SDNN values decrease significantly in the high-strain/stress group [46]. The sharp decrease in the SDNN causes significant tension of regulatory systems of the body, which leads to the suppression of the activity of an independent contour to the stress [47]. In [48], authors noticed that special attention should be paid to the evaluation of the SDNN, which is an integral indicator characterizing the HRV as a whole for the recording period and depends on the impact of both sympathetic and parasimpathic nervous systems. The SDNN correlates to the total HRV power. It has been established by physicians that a low HRV value can imply increased fatigue, which can be associated with increased stress [49]. After all HR data were collected, the spikes (outliers) were deleted and the HRV was calculated according to Equation (1), where N is the total number of the heart beats in the time window:

(1) $$\mathrm{SDNN}=\sqrt{\frac{1}{N-1}\sum_{n-2}^{N}{\left(RR_{j}-RR_{avgj}\right)}^{2}}$$

For calculation we used 30 and 60 s intervals, both calculated SDNN values were chosen as attributes in the machine learning algorithms. It should be noted that the recommended lengths used in the HRV analyses are 5 min for short-term analysis and 24 h for long-term. Nevertheless, some research [50] has shown that a good correlation was found between the 1 min and the 5 min parameters for the maximal-RR (maximal inter-beat interval), the minimal-RR (minimal inter-beat interval), the average-RR (average inter-beat interval), SDNN, RMSSD (root mean square of successive differences), pNN50 (a measure of the number of consecutive NN intervals which differ by more than 50 ms), and for the total power.

### 3.3. GPS Data

The GPS data is objective data, which can be used for the classification of the driving style. To collect the GPS data, two sensors were used: one is a built in GPS smartphone sensor (driver’s smartphone was placed on the dashboard inside the car) and the second one is a U-blox GPS receiver with an external active GPS/GLONASS antenna on the car roof. The U-blox GPS sensor was used as a reference.

Using the smartphone, we got very similar GPS data as we did with the U-blox GPS receiver, with the difference of a lower resolution of 1 Hz in the former case. During the experiment, the smartphone GPS sensor was found to be more convenient for the drivers, because they did not need to set up an external antenna and charge the devices.

**Figure 3:**
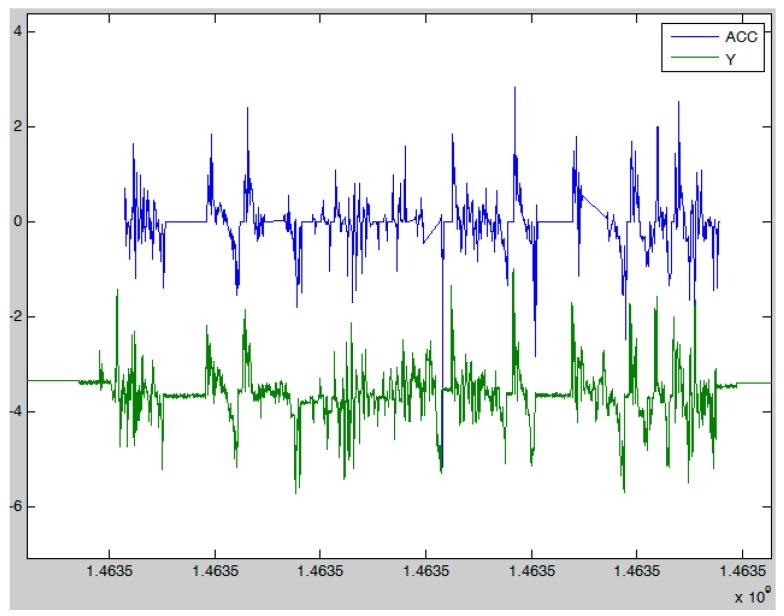
Acceleration calculated from GPS data and Y axis values from smartphone accelerometer.

To recognize aggressive driving we also compared acceleration calculated from the U-blox GPS data to the data received from the accelerometer in the smartphone and found a very strong correlation. In Figure 3, we present the acceleration data that is gathered from the accelerometer built-in smartphone (Y) and calculated with the U-blox GPS receiver speed data (ACC). The acceleration data was used when the GPS signal was lost due to bad reception (in tunnels, etc.).

### 3.4. The Car Door Data

The car door is the first “system”, with which the driver interacts when starting a drive. An Android smartphone for collecting the car door opening and closing data was placed in the car door. Our assumption was that adding the car door data to the analysis model will improve the accuracy of driving style prediction, as it may indicate that the driver is in a hurry.

**Figure 4:**
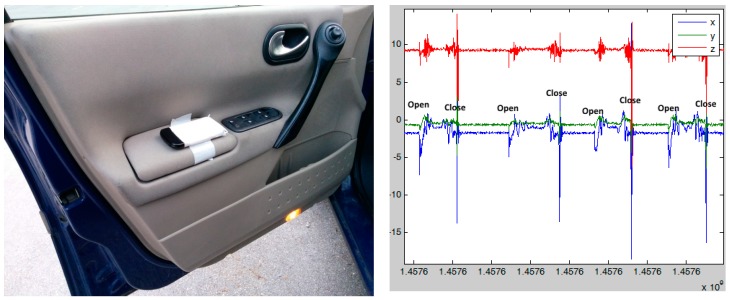
An Android smartphone for collecting of car door data (**left**) and example of these data (**right**).

In Figure 4, a smartphone in the car door and raw data of the car door opening and closing are depicted; there are opening and closing actions and the time between the opening and closing the car door. These features were added to the analysis model.

The sampling rate of the accelerometer on a car door was 40 Hz, which was enough to get certain features from the car door accelerometer:

- Time between opening and closing the car door;
- Time between opening the car door and starting the engine;
- Intensity of the car door movement (how fast one opens/closes the car door).

The data was cleaned in the sense that the acceleration spikes, which occurred on rare road bumps, were ignored and only data from real door closing/opening was used (in standstill).

### 3.5. Description of the Experiment

The experiment was designed to test a driving style in general for all drivers. For this purpose, 10 participants were involved in the experiment, five males and five females aged from 30 to 45 years. All were employed and drive to work every morning and back home in the afternoon. For most of the drivers the experiment lasted one week. Each participant made 14 trips on average. The dataset from one participant was not acceptable due to a big amount of missing data, which was due to technical issues. In total, 123 driving trips were made by nine drivers resulting in approx. 2.6 million rows of data in the database. The research kit includes:

- An Android smartphone for collecting the car door data. A handmade holder with a mobile phone is depicted in Figure 4 (during the experiment the phone was permanently fixed in the car door in the same position);
- The other smartphone in the experiment was always the participant’s private mobile phone in order to be able to collect his/her environment and activity data with sensors embedded into a smartphone, and to avoid using additional devices;
- A car—each participant’s private car;
- The Polar H7 chest belt (Polar Company, Kempele, Finland). The Polar H7 was used as a reliable sensor for collecting the HR data in many researches [51,52], serving as the ground truth data for estimation of the driver’s stress level [53].
- A U-blox GPS device for gathering speed and acceleration data.

We asked the participants to follow their regular routes and habits, and not to record the driving during a heavy rain to avoid driving in adverse weather conditions, which could lead to unusual driving [54]. The average trip duration was 34 min (with median of 26 min).

We should note that we experienced a significant loss of HR data due to poor BT connectivity and conflicts with other BT devices in a car. Therefore the HR rate data was omitted from the experiment.

### 3.6. Procedure

The experimental scenario, which was used for data collection from the 10 participants, is presented below. The participation in the experiment was voluntary and the participation agreement was signed by every participating driver. This experiment was designed to, as seamlessly as possible, capture driver’s activity and environment data in real-life scenarios before and during the driving. The various road situations and stressors were not controlled; drivers were able to use their usual routes. Most of the routes included city and highway roads—all of them were asphalted.

**Figure 5:**
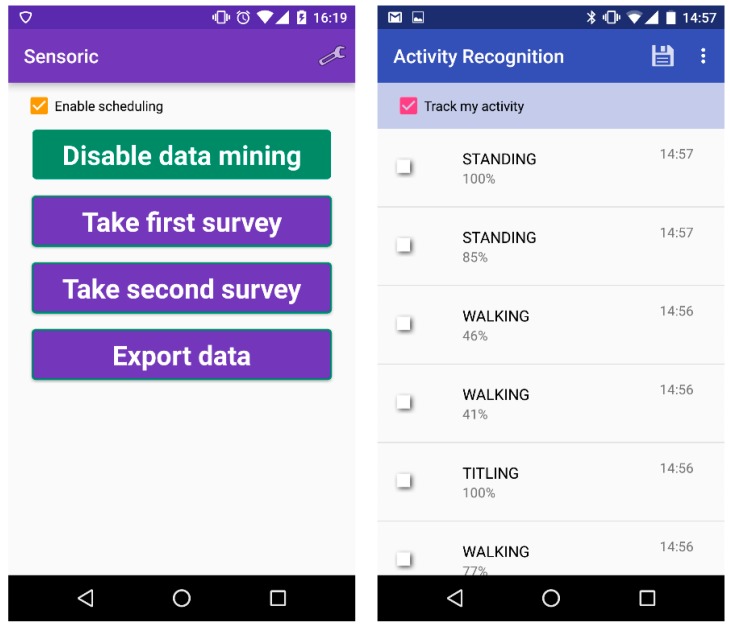
Sensoric application for collecting smartphone data and android application for activity recognition based on the GAR API. (**left**) main menu; (**right**) activity recognition subscreen.

The procedure started approximately 1 h before the driving, when the participant took the first survey and turned on the data gathering process through the *Sensoric* application [55]. This triggered the application on the phone in the car door through an SMS, which started collecting the accelerometer data. The participant was asked to put on the heart rate chest belt and start recording the HR data 10–15 min before the driving to get adapted to the chest belt. In a car, we used the participant’s smartphone to collect the GPS data, the HR data and the *Sensoric* data. After the driving, participants took the second survey, which included the assessment of traffic and of their driving style, which led to the automatic switch off of all data gathering, both on the participant’s phone as well as on the car door phone. The main screens of the developed application *Sensoric* and Google activity recognition is depicted in Figure 5.

## 4. Results and Discussion

In the study we were aiming at the evaluation of the drivers’ self-assessment regarding their driving style and at the prediction of the driving style, using the objective environment and activity data. The self-assessment evaluation was implemented through a self-reporting questionnaire, filled in after driving, and its comparison to the objective driving data. As is shown in this section, the results indicate a significant level of drivers’ subjectivity when it comes to self-assessment of their driving style.

The second metric, which was developed to estimate the influence of the users’ environment and the drivers’ activity information to the driving style, was based on the amount of the aggressive acceleration, braking and turning, normalized by the driving time. We were able to successfully recognize aggressive driving before it actually took place using only the smartphone data. Due to the significant loss of HR data we had to omit the third foreseen metric, namely the influence of stress level to the aggressive driving.

### 4.1. Driving Style Self-Assessment

The driving style self-assessment is based on the 7- point Likert scale filled out after driving. The objective data, measured through GPS and accelerometer sensors has been used before in similar research to define aggressive driving [11,43,55,56,57]. The threshold for aggressive driving, which includes acceleration, breaking and turning was set at 2.5 m/s^2^. This was done based on visual data analysis and successful previous researches [55,56,57].

When compared to the objective driving data, obtained from the GPS and accelerometer sensors, the regression analysis results in the correlation coefficient of 0.58, while the correlation coefficient for male and all drivers is even lower (overall correlation coefficient is equal to 0.51). We are attributing these poor results to the unreliability of the self-assessment approaches as people can be subjective and can over- or underestimate their actions (driving in our case).

Filling the questionnaire after driving can also cause non-objectivity because the results depend on the drivers’ memory, attention and mood influenced by cognitive distortions or simply by the desire to embellish their true driving style. Anyhow, we can conclude that the self-assessment was not a successful approach in our study.

### 4.2. Prediction of the Driving Style—Full Data Set

For the prediction of the driving style, we first used the environment influence data, physiological and drivers’ activity data collected before driving, while additional analysis included also the car door data and the data of the first 1 minute of driving. The ground truth for definition of aggressive driving was defined using objective acceleration, breaking and turning data as described in Section 4.1:

(2) $$F-Measure=2\times \frac{Precision\times Recall}{\left(Precision+Recall\right)}$$

For evaluation of the results one of the standard measures of a test’s accuracy was used: F-measure (Equation (2)). The F-measure is a combined measure for precision and recall; it is a balanced mean of precision and recall. The recall is a number of examples predicted positive that are actually positive. The precision is a proportion of the examples, which truly have the class X divided by the total, classified, as the class X.

The best machine learning algorithms used for classification were Bayesian Networks (BN) and SMO (Sequential Minimal Optimization). BN is a probabilistic statistical model, which represents a set of random variables and their conditional dependencies via a directed acyclic graph [58]. SMO is an efficient method for training support vector machines, which aim at classification of data in a high-dimensional space, where the largest margin between classes can be found [58].

For the analysis, we first applied the standard Ranker search method, providing the feature selection from the complete feature set, described in Section 3.1. The features selected by the Ranker search method were intensity of car door closing (x and y axis), median values of smart phone gyroscope 5 to 15 min before driving, number of screen on/off states in last 30 min before driving, number of hard accelerations during 1st min of driving (above 2.5 m/s^2^) and median of light and noise deviations 10 min before driving. The data were grouped into 3 classes: the “aggressive” driving, the “normal” driving, and the “moderate” driving, based on the threshold defined in Section 4.1 and Section 4.2. 11 trips belong to “aggressive” class, 10 trips belong to “moderate” class and 94 trips belong to “normal” class. We were mostly interested in recognition and correct classification for the “aggressive” driving class.

**Figure 6:**
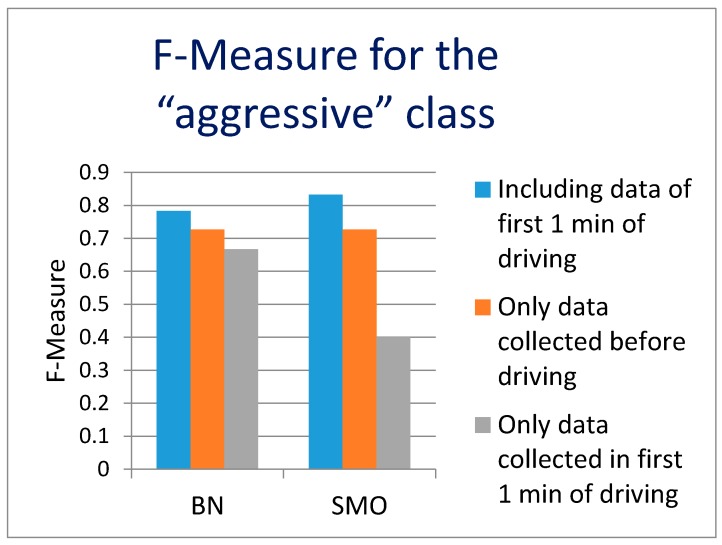
The F-Measure of the classification aggressive driving (“a” class) for the data collected before the driving and for the data collected before and during the first 1 min of the driving.

**Figure 7:**
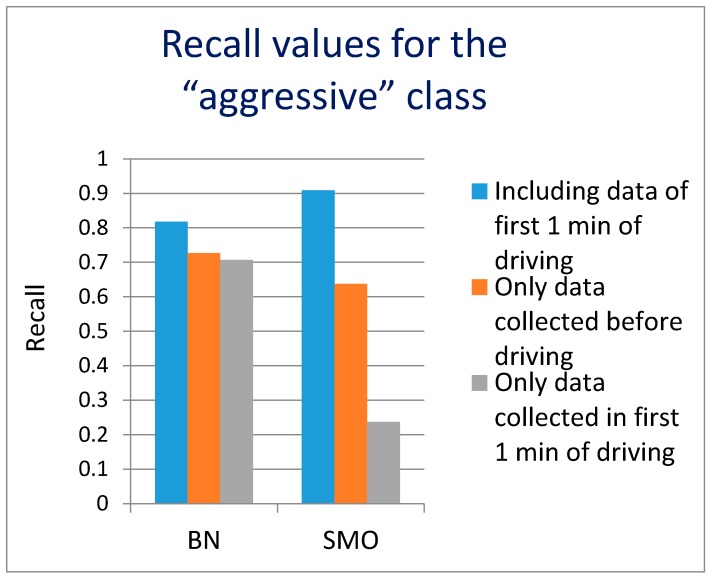
The values of recall for aggressive driving for different data sets and algorithms.

The first analysis results are presented in Figure 6 (F-measure for “aggressive” class) and Figure 7 (recall for “aggressive” class). The F-measure value of 0.727 (0.727 precision, 0.727 recall) was achieved using both classifiers. If the first 1 min of the driving data is included in the analysis, the F-measure value increases to 0.833 (0.769 precision, 0.909 recall) using SMO and somewhat lower value for BN. It should be noted however that using only the data from the first 1 min of driving to predict the later overall driving style, leads to weaker results F-measure 0.667 using BN and even lower value of 0.4 using SMO. This seems understandable as the time window of the first 1 min of driving usually consists of warming up the engine, buckling up and driving out of a parking lot, a garage, etc. Therefore it is not really a typical representative of later driving. However, we believe that if the driver accelerates or brakes aggressively at the very beginning of driving (first 1 min), there is a high probability he/she will drive aggressively even further. The data from the first 1 min of driving does not bring useful results on its own, but additionally improves the results when combined with environment data. It leads us to the conclusion that the environment data, collected before driving, is relevant and enables the prediction of the driving style. We believe that the recall values are more important than precision, because a higher recall means that more of potentially aggressive driving can be recognized (9 out of 10). On the other hand, if occasionally some non-aggressive drivers are warned (2 out of 10), not much damage will be done.

### 4.3. Prediction of the Driving Style—Personal Smartphone as the Only Data Source

**Figure 8:**
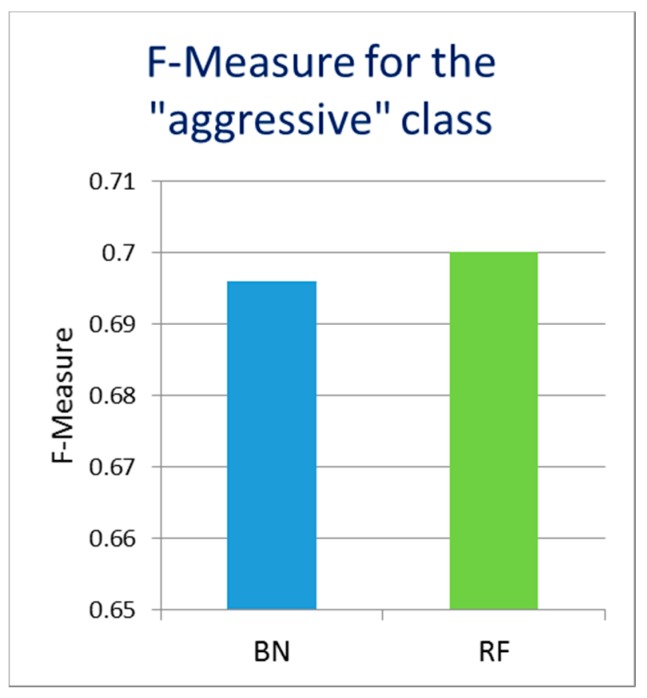
F-Measure of classification of aggressive driving for the data collected with only smartphone before the driving, without using the car door data.

Finally, we evaluated the possibility of usage and sufficiency of the users’ environment data received from a personal smartphone as the only data source to predict the driver’s driving style (without the car door data). The results for the F-Measure value for the “aggressive” class are presented in Figure 8, where RF presents the random forest algorithm and BN presents Bayesian networks algorithm.

The underlying recall values in this case achieved the level of recall values when the car door data were used for the classification, while at the same time the precision values were lower, resulting in a lower F-measure (around 0.7). Therefore, the precision of identification of the “aggressive” class without the car door data is slightly reduced, but not dramatically. Therefore, it seems possible to estimate the driving style in advance even before driving, using only the data collected by the smartphone. This seems convenient because most people always carry a smartphone with them. The inclusion of car door data though, adds to accuracy of the predictions and are a good addition for the prediction of the driving style.

### 4.4. Limitations of the Study

As the study included 10 test persons aged 30–45, there some limitations to the interpretation of the results. Although the age group of 30–45 year olds is one of the most representative ones, we should not jump to conclusions that the results can be uncritically extended to other age groups without additional tests and experiments. The number of participants is sufficient for an initial pilot study, but additional participants of other age groups should be included in later studies, confirming the wider scope of results.

## 5. Conclusions and Future Work

New models and methods were designed to estimate the influence of the users’ environment and the drivers’ activity information to the driving style in a usual automotive environment during the natural driving. This is an advantage of this research, because the experiment was conducted in real-life scenarios, not in a simulation or pre-described environment. The obtained results lend support to the hypothesis that users’ environment and activity data can be used for the prediction of driving style, getting us an estimation, before the actual driving takes place.

In the experiment, a data gathering system was developed for prediction of the driving style. The design of the driving style estimation experiment was built upon the findings of both self-assessment of the driving style experiment and the experiment for estimation of the driving style based on the objective driving data. The system uses the users’ environment data and information about the activity of the drivers captured by two smartphones, one of them being placed in the car door. This analysis incorporates several previously developed features as well as two new types of features: a car door opening and closing manner and recognition of the current type of activity before the driving using the GAR.

The analysis of the estimation of the driving style based on drivers’ self-assessments showed a low overall correlation coefficient equal to 0.51. We attribute this to the fact that people can be subjective in their self-assessments and can over- or underestimate their driving style.

For the driving style prediction based on gathered users’ environment and activity data a number of tests were conducted. Results show that the inclusion of the first 1 min of the driving data (also including the HR data) aggressive driving could be recognized with 0.909 recall and 0.769 precision values, F-measure is 0.833. In this case, the recall value is more important, because the recall shows the share of truly aggressive driving styles predicted, which is a desirable feature allowing for early warnings, etc. Using only the data collected before driving and including the car door opening and closing manners, the prediction of aggressive driving has achieved values of 0.727 for recall and 0.727 for precision. Using only the data collected before driving even without car door data, the precision drops further to 0.667. Therefore the inclusion of the first 1 min of the driving data improves the prediction, as does the inclusion of the car door data, while using this data on their own does not result in good predictions.

Suggested usage of the driving style prediction could be applied in non-critical applications such as automatic management of different applications, e.g., calls, SMS, messengers, navigation aids, or as an evaluation tool for the road or as the application for those who want to improve their driving style, fuel consumption and CO_2_ emission. This is based on the fact that the increments of the fuel consumption and CO_2_ emissions have a strong positive correlation with aggressive driving, +0.856 [57]. Furthermore, the business models could include self-improvement applications for drivers or more robust driving applications issued by insurance companies, which are already offering the PHYD (pay how you drive) model.

Based on the promising results of the pilot study, the future work, in our opinion, should include: the standardization of aggressive driving description and parametrization, gathering of more driving data to test obtained conclusions on a wider sample of drivers and, checking of the influence of automated warnings issued to drivers on changes in the driving style. With a wider sample of the driving data it could be possible to build a general model for the driving style prediction as well as individual models for different drivers. In such a system the typical beginning of system usage (no knowledge about the user), would include the general model for all drivers, probably customized to a certain age group, but later, when enough data has been collected, build the individual analysis models for each driver. The current work shows that the driving style prediction through the analysis of the data collected before driving in a non-invasive way is becoming possible. The identified contributions should be helpful in getting closer to such car systems, which can respond intelligently to the driver’s behaviour. Transforming such a system into a real-time solution, would require sending the data to the server, where the data would be analyzed and sending the identified warnings back to the smart phone. Processing the data on the smart phone itself seems an attractive proposition, but would currently require too much processing power and battery life for practical purposes.

Making the cars and the world’s roads safer is a critical component of saving lives on the roads including the legislation related to the reduction of road traffic injuries and deaths. In addition to these very important components of safe driving, it is also possible to use suggested self-coaching apps to improve the current driving style, at least for people who are willing to accept it.
